# New tools to measure community and stakeholder engagement and its impact on outcomes of clinical research

**DOI:** 10.1186/1742-4690-9-S2-O24

**Published:** 2012-09-13

**Authors:** P Bahati, S Hannah, S Seidel, S Aggett, R Siskind, R Campbell, S Williams, A Downie, S Rosas

**Affiliations:** 1 International AIDS Vaccine Initiative, Nairobi, Kenya; 2 AVAC, New York, NY, USA; 3 Global Alliance for TB Drug Development, New York, NY, USA; 4 Wellcome Trust, London, UK; 5 National Institute of Allergy and Infectious Diseases, USA; 6 Fred Hutchinson Cancer Research Center, USA; 7 International HIV/AIDS Alliance, UK; 8 Concept Systems, Ithaca, NY, USA

## Background

Community and stakeholder engagement have increasingly been acknowledged as best practice in the design and implementation of global clinical research, results dissemination and strategies for access to new health products. Existing guidelines and best practices, however, provide little insight into the expected outcomes of engagement activities, indicators of success, or useful monitoring and evaluation (M&E) tools for assessing impact on research and communities.

## Methods

To fill this gap, a consortium of organizations (AVAC, HANC, International HIV/AIDS Alliance, IAVI, NIAID, TB Alliance and Wellcome Trust), developed and field tested a user-friendly M&E Toolkit for engagement programs in clinical research settings in developing countries. From a broad review across clinical trial settings, the toolkit builds on existing practices and introduces new methods for M&E, indicators for impact and guidelines for effective use. The toolkit was piloted with TB and/or HIV research sites in Africa and Asia that have a history of incorporating engagement strategies in research.

## Results

New indicators for measuring the degree of engagement and its impact on clinical research were identified. Participants in pilot-testing utilized existing and new methods of evaluation, and assessed the relationship of these strategies on targeted outcomes of clinical research. Feedback and lessons learned are being incorporated into a final version of the M&E Toolkit.

## Conclusion

Understanding the impact of stakeholder engagement on clinical research is critical for the development and implementation of effective strategies, and for planning and optimizing clinical trials and their outcomes in developing countries. Through the use of the toolkit site and sponsor staff can better evaluate strategies and activities, demonstrate benefit, reallocate resources to activities that were most productive, and make the case for engaging stakeholders in research.

